# Acute effects of re-warm-up on physical performance, muscle temperature, and perceived exertion in basketball players: a systematic review and meta-analysis with individual participant data

**DOI:** 10.3389/fphys.2026.1821278

**Published:** 2026-07-08

**Authors:** Yu-Feng Yang, Enrique Flórez-Gil, Christos Koutsouridis, Yu-Ming Zhong, Nuno Mateus, Eduardo Abade, Ching-Feng Cheng, Mengde Lyu, Kai Xu

**Affiliations:** 1 School of Physical Education, Shanghai University of Sport, Shanghai, China; 2 Faculty of Physical Activity and Sports Sciences, VALFIS Research Group, Institute of Biomedicine (IBIOMED), Universidad de León, León, Spain; 3 Aristotle University of Thessaloniki, School of Physical Education & Sport Science, Laboratory of Evaluation of Human Biological Performance, Thessaloniki, Greece; 4 School of Medical and Health Sciences, Centre for Human Performance, Edith Cowan University, Joondalup, WA, Australia; 5 School of Athletic Performance, Shanghai University of Sport, Shanghai, China; 6 Research Center in Sports Sciences, Health Sciences and Human Development, CIDESD, Elite Research Community, Vila Real, Portugal; 7 Department of Sports Science, Exercise and Health, School of Life Sciences and Environment, University of Trás-os-Montes and Alto Douro, Vila Real, Portugal; 8 Portugal Football School, Portuguese Football Federation, Oeiras, Portugal; 9 Department of Sport and Kinesiology, National Taiwan Normal University, Taipei, Taiwan; 10 Sports Performance Lab, Department of Sport and Kinesiology, National Taiwan Normal University, Taipei, Taiwan; 11 School of Biomedical Science and Health, Royal Melbourne Institute of Technology University, Melbourne, VIC, Australia

**Keywords:** re-warm-up, physical performance, half-time, rating of perceived exertion, body temperature

## Abstract

**Objectives:**

(1) the acute effects of RWU on physical performance, body temperature (BT), and rating of perceived exertion (RPE) in basketball players; and (2) individual-level performance improvements across different RWU methods.

**Methods:**

Three databases were searched for RWU studies in basketball players. An individual participant data meta-analysis was conducted using a two-stage approach with multilevel models, including adjustments for small sample sizes. Effect sizes were expressed as standardized mean changes (SMC) for within-group comparisons and standardized mean differences (SMD) for between-group comparisons.

**Results:**

Seven studies (n = 82; 35 females, 47 males) were included, with individual raw data obtained from three. Compared with the control condition, the RWU resulted in better preservation of jumping (SMD = 0.64) and change-of-direction (COD) performance (SMD = −0.83), while showing no significant differences in isometric mid-thigh pull (IMTP) peak force (SMD = 0.34), BT (SMD = 0.72), or RPE (SMD = 0.61). These effects were largely driven by greater declines in the control than in the RWU for jumping (SMC = −1.24 vs. −0.53), COD (SMC = 1.00 vs. 0.44), and IMTP peak force (SMC = −0.68 vs. −0.28). Across five interventions from three studies with individual data, the plyometric protocol showed the highest proportion of improvements (CMJ: 100%; COD: 93.3%) compared with the control condition.

**Conclusions:**

Preliminary evidence suggests that RWU may be an effective strategy to maintain physical performance and BT in basketball players. However, given the limited number of studies and the high degree of individual variability, further research is required.

## 1 Introduction

Basketball is a mixed-metabolic sport that combines aerobic and anaerobic demands, requiring players to perform frequent bouts of running, jumping, changes of direction, and physical contact throughout a game (Ramirez-Campillo et al., 2022; Devesa et al., 2024; Flórez-Gil et al., 2025a; Tian et al., 2025). Prior to the start of competition, players typically perform an initial warm-up to facilitate readiness and enhance performance, thereby meeting the high-intensity demands of the game (Silva et al., 2018; Koutsouridis, 2023; Flórez-Gil et al., 2025b; Yang et al., 2025). However, not all players are able to enter the game immediately after the initial warm-up (Flórez-Gil et al., 2025b). Some players, such as non-starters, may remain seated following the warm-up, which could lead to a decline in performance. For example, a study involving basketball players reported that during the passive period following the warm-up, body temperature (BT), jumping ability, and sprint performance decreased rapidly (Galazoulas et al., 2012; Crowther et al., 2017). Specifically, BT gradually declined from 36.9 °C to 36.2 °C over a 40-minute passive recovery period. Meanwhile, jump performance decreased from 13% at 10 minutes to 20% at 40 minutes, and 20-m sprint time increased from 3.9% at 10 minutes to 6.3% after 40 minutes of rest (Galazoulas et al., 2012). Similarly, during the halftime interval, which typically lasts about 15 minutes, players who remain seated may also experience performance decrements (Russell et al., 2015; Xu et al., 2025a).

Accordingly, re-warm-up (RWU) strategies in basketball are typically implemented in two situations: (1) after the initial warm-up but before entering the game (e.g., for non-starters) (Flórez-Gil et al., 2025b), and (2) during the halftime interval (Pociūnas et al., 2018; González-Devesa et al., 2022; González-Devesa et al., 2023; Koutsouridis et al., 2024, 2025; Yang et al., 2025). Under these two scenarios, RWU may represent an effective strategy to prevent reductions in BT and physical performance. For instance, Flórez-Gil et al. (2025b) reported that incorporating a series of plyometric jump exercises after the initial warm-up effectively maintained countermovement jump (CMJ), drop jump (DJ), and change-of-direction (COD) performance. Similarly, Koutsouridis et al. (2024) showed that a 3-minute cycling RWU at 40% of maximal oxygen uptake during halftime helped maintain CMJ and COD performance, as well as BT. Consistently, other studies have also demonstrated positive effects of RWU strategies on CMJ (Pociūnas et al., 2018; Koutsouridis et al., 2024; Yang et al., 2025) and COD performance (Yang et al., 2025).

However, inconsistent findings have been observed. For example, González-Devesa et al. (2022; 2023) reported that neither the RWU strategy nor passive rest produced significant changes in CMJ performance, and that a short, high-intensity RWU resulted in significantly higher ratings of perceived exertion (RPE) compared with passive rest. These discrepancies may be attributed to differences in RWU modalities (RWU after initial warm-up and halftime RWU), including type, duration, intensity, and the performance measures used to assess outcomes (Bishop, 2003; Xu et al., 2025a). Taken together, these findings indicate that the effects of RWU in basketball remain inconclusive. Considering that RPE is commonly used as a supplementary indicator of internal load to reflect subjective exercise strain during RWU strategies, changes in RPE may therefore provide complementary information regarding the acute physiological and perceptual responses to different RWU protocols (Eston, 2012).

To date, only one review has qualitatively examined the effects of RWU on physical performance in basketball players, and the limited number of included studies makes it difficult to draw robust conclusions (Devesa et al., 2024). Compared with conventional aggregate data meta-analyses, an individual participant data meta-analysis allows for more precise estimation of intervention effects and enables the examination of inter-individual variability, including responder and non-responder profiles across different RWU strategies (Tierney et al., 2019). To strengthen the level of evidence, the present study aims to obtain original data and conduct a quantitative investigation using an individual participant data meta-analysis. Specifically, this study seeks to address the following two questions: (1) the acute effects of RWU (including all RWU strategies and halftime-only RWU) on physical performance, BT, and RPE in basketball players; and (2) the magnitude of performance improvements and their percentage changes at the individual level across different RWU methods.

## 2 Methods

### 2.1 Systematic review protocol registration

This systematic review followed the Preferred Reporting Items for Systematic Reviews and Meta-Analyses of Individual participant data (PRISMA-IPD) 2015 guidelines (Stewart et al., 2015). The study protocol was preregistered in the Open Science Framework (osf.io/wfvny/overview) before data analysis.

### 2.2 Eligibility criteria

Inclusion criteria were as follows: (1) Participants: basketball players of at least McKay et al. (2022) Tier 2 (i.e. trained individuals), of any age and sex; (2) Intervention: RWU protocols performed after the initial warm-up (before the start of the match) or during half-time (after two quarters of play), typically conducted over a short duration (e.g., < 5 min); (3) Comparison: passive rest or low-intensity activities (e.g., shooting practice); (4) Outcomes: at least one physical performance-related measure (e.g., jumping, COD), BT and RPE were considered secondary outcomes and were extracted when available; (5) Study design: randomized, non-randomized, crossover, or parallel trials; (6) Publication type: peer-reviewed original articles published in English.

For eligible original studies, the corresponding authors were contacted via email to request the raw data. If no response was received, a follow-up email was sent one week after the initial contact, and this process was repeated twice, given that this was an individualized analysis.

### 2.3 Search strategy and study selection

Searches were conducted in three English databases (Web of Science, SPORTDiscus, and PubMed). Additional relevant studies were identified by reviewing published RWU reviews, utilizing Google Scholar, and citations tracking of included studies. The search was independently conducted by two reviewers (Y.M.Z. and K.X), covering all records from database inception through November 20, 2025. Boolean phrases and keywords were as follows: (“re warm up”) AND (“basketball” OR “non started” OR “half time”). For details, see **Appendix 2** of the **Supplementary Materials**. As no unpublished studies meeting the topic criteria were identified during the subsequent searches, no unpublished data were included in this review.

One reviewer (K.X.) initially screened titles and abstracts to identify potentially relevant studies. Full-text screening was then conducted independently by two reviewers (Y.M.Z. and K.X.) based on predefined inclusion and exclusion criteria. Discrepancies or uncertainties were resolved through consultation with a third reviewer (H.X.C). The entire screening process was conducted using EndNote reference manager (version 21; Clarivate Analytics, Philadelphia, PA, USA).

### 2.4 Data extraction

Data extraction was independently conducted by one reviewer (K.X) and validated by two others (Y.M.Z. and H.X.C). Extracted data were organized in Excel^®^ (Microsoft Corporation, Redmond, WA, USA) under the following categories: i) Basic information: authors, publication year, sample size, sex, age, height, body mass, training experience, and competitive level; ii) Details of the RWU and control conditions: modality, content, intensity, and duration. ii) Outcomes: jump performance (e.g., CMJ and DJ), COD performance, isometric midthigh pull (IMTP) peak force, BT, and RPE. iii) Measurement time points during the intervention and the direction of outcomes. Three studies presented as images were extracted using WebPlotDigitizer (version 4.5; https://www.colliseum.net/WebPlot/).

### 2.5 Risk of bias evaluation and certainty assessment

Risk of bias was assessed using the Cochrane Risk of Bias tool (RoB-2) (Sterne et al., 2019). Two reviewers independently evaluated five domains. Evidence certainty was rated using the Grading of Recommendations Assessment, Development and Evaluation (GRADE) system (Schünemann et al., 2019). Evidence initially received a high certainty and was downgraded based on the following criteria: small sample size (≤ 400), substantial heterogeneity (*I²* > 50%), unclear direction of the pooled effect, and risk of publication bias (Singh et al., 2023; Zhu et al., 2026). All assessments were also independently conducted by two reviewers (Y.M.Z. and K.X.), with any disagreements resolved through discussion.

### 2.6 Statistical analysis

Based on the three studies (Koutsouridis et al., 2024, 2025; Flórez-Gil et al., 2025b) for which individual participant data were obtained, pre–post correlation coefficients (r) were first calculated for jump height (including CMJ and DJ), COD, BT, and RPE. These coefficients were subsequently transformed using Fisher’s z. For studies reporting multiple effect sizes or comparisons, multilevel models were applied, whereas studies with a single comparison were synthesized using conventional two-level models. The resulting estimates were then back-transformed to *r* and used to supplement studies without available raw data (Robinson et al., 2024).

The final estimated pre–post correlations for the RWU group were 0.876 for jump height, 0.945 for COD, 0.800 for BT, and 0.400 for RPE. The corresponding values for the control group were 0.891, 0.830, 0.400, and 0.800, respectively. For IMTP peak force, where no raw data were available, a common correlation coefficient of 0.870 (applied to both the RWU and control groups) was imputed based on the average correlation across all performance outcomes.

As jump performance was assessed using multiple test types [e.g., both CMJ and DJ in Flórez-Gil et al. (2025b)], a clustering coefficient (*ρ)* required for cluster-robust variance estimation was calculated using the available raw data, following the method proposed by Hedges et al (Hedges et al., 2010). The estimated value of *ρ* was 0.25 and was used to compute pooled effect sizes for both within- and between-group comparisons of jump performance. This approach, based on borrowing information from comparable outcomes, helped reduce the need for extensive sensitivity analyses.

The primary analyses pooled the effects of RWU (including both post–initial warm-up and halftime RWU) on jump performance, COD, and IMTP peak force. Where sufficient data were available, subgroup analyses were conducted for jump performance, separately examining CMJ, DJ, and squat jump (SJ). To isolate the effects of halftime RWU, additional analyses were performed after excluding the study by Flórez-Gil et al. (2025b), which investigated RWU following the initial warm-up.

Given that the benefits of RWU may largely reflect the maintenance of performance, within-group pre–post changes were also presented separately for the RWU and control conditions (Xu et al., 2025b, 2025a). Similar analyses were conducted for BT and RPE. In addition, results were reported for both the entire simulated match period and the halftime-only RWU condition. All pooled results were adjusted for small sample sizes (Pustejovsky and Tipton, 2022; Jukic et al., 2023). Finally, based on the three studies with available raw data, individual-level percentage changes in performance were calculated for the intervention group relative to baseline. Additional analyses were conducted to determine percentage changes relative to baseline after accounting for the control condition.

Standardized mean change (SMC) was used for within-group comparisons, and standardized mean difference (SMD) was used for between-group comparisons. As this study was exploratory in nature, effect sizes were not categorized according to magnitude. Heterogeneity was evaluated using *I²*, *τ²*, and Q statistics, with *I²* values of 25%, 50%, and 75% indicating low, moderate, and high heterogeneity, respectively. These indicators reflect both relative and absolute residual heterogeneity, representing unexplained variability. Prediction intervals (PI) were calculated to better account for the potential variability of similar studies in the future. Heterogeneity was modeled using a mixed-effects model with study as a random effect. For multilevel models, total heterogeneity was obtained by summing the level-2 and level-3 variance components.

Funnel plots were generated for both within- and between-group comparisons to assess publication bias. Given the small number of studies (*k* < 10), Egger’s test was not performed. All analyses were conducted in R (version 4.5.2; R Core Team, Vienna, Austria) using the *metafor* package (Viechtbauer, 2010), with robust and small-sample adjustments implemented through the *clubSandwich* package (Pustejovsky and Tipton, 2022). Pooled estimates were extracted using the *orchard* package (version 2.0) (Nakagawa et al., 2023), while forest and funnel plots were created using the *ggplot2* (Wickham, 2011)and *metaviz* (Kossmeier et al., 2020) packages, respectively. Figure assembly was performed using the *patchwork* package (Pedersen, 2019).

## 3 Results

### 3.1 Search results and study characteristics

**Figure 1:**
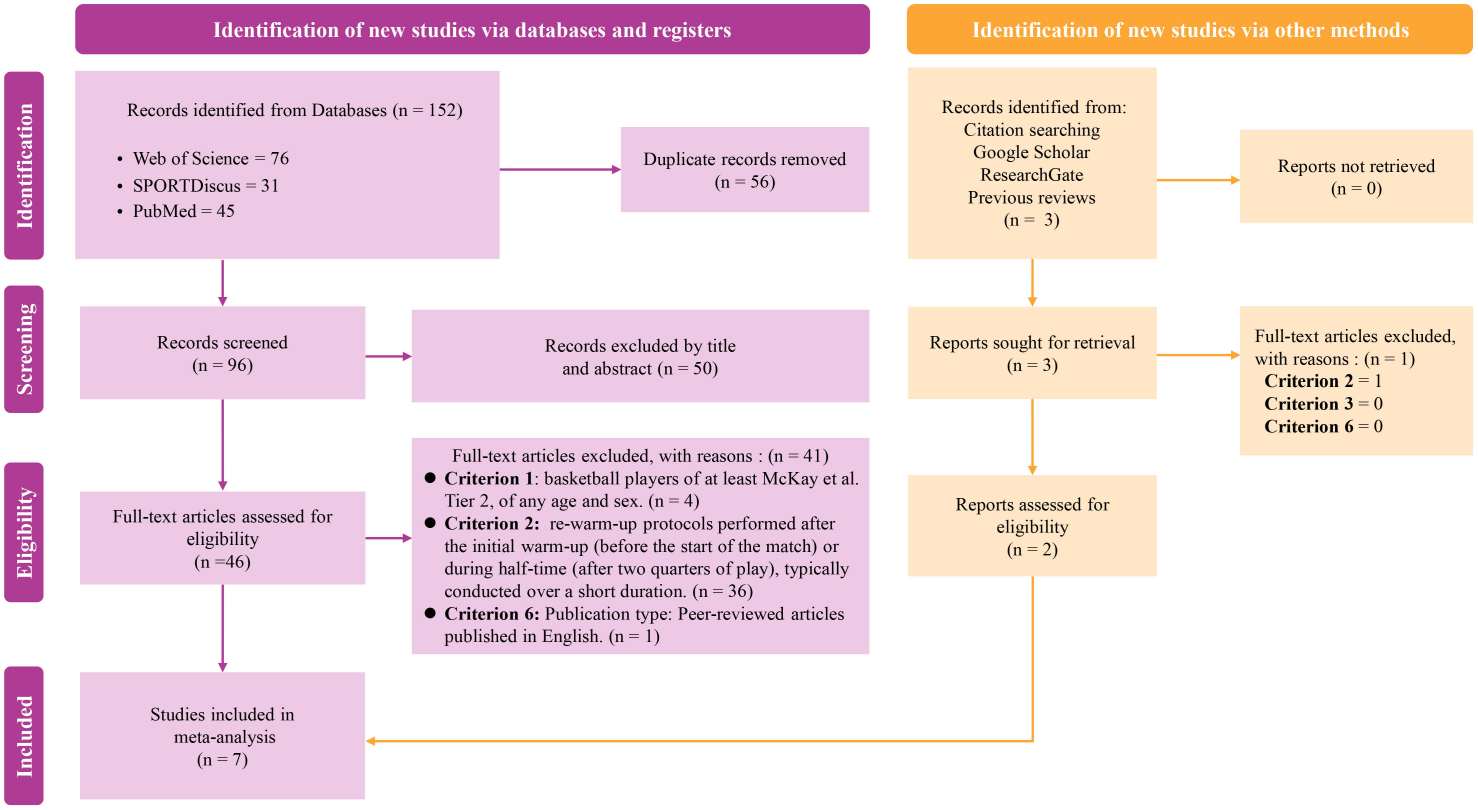
PRISMA flow diagram detailing the study inclusion process.

A total of seven studies (Pociūnas et al., 2018; González-Devesa et al., 2022; González-Devesa et al., 2023; Koutsouridis et al., 2024, 2025; Flórez-Gil et al., 2025b; Yang et al., 2025) were ultimately included (**Figure 1**). The corresponding authors of all studies were contacted by email: three provided the requested raw data (Koutsouridis et al., 2024, 2025; Flórez-Gil et al., 2025b), one declined the request (Yang et al., 2025), and three did not respond (Pociūnas et al., 2018; González-Devesa et al., 2022; González-Devesa et al., 2023).

In total, 82 basketball players were included (female: n = 35; male: n = 47). One study involved trained athletes (Tier 2), whereas the remaining six studies involved highly trained athletes (Tier 3). Participants’ ages ranged from 13.7 ± 0.8 to 22.0 ± 5.0 years. Body mass ranged from 54.0 ± 14.0 to 86.0 ± 5.5 kg, height ranged from 1.62 ± 0.11 to 1.93 ± 0.10 m, and training experience ranged from 4.2 ± 1.1 to 9.5 ± 2.9 years.

Regarding study design, one study implemented a RWU after the initial warm-up, four studies reported only the performance changes following the RWU, and two studies reported performance changes after the initial warm-up, after the third quarter, and after the fourth quarter. The RWU protocols included plyometric jumps, sprinting, cycling at either 40% or 80% of maximal oxygen uptake, bodyweight-based dynamic exercises, and core strength training.

Regarding outcome measures, all seven studies reported CMJ height. One study reported SJ height, one reported DJ height, four reported COD performance, and one reported IMTP peak force. Additionally, three studies reported RPE, and two studies reported BT (**Table 1**).

**Table 1:** Study characteristics.

Study	Participants	Re-warm-up	Measure	Results
Pociūnas et al. (2018)	10M Trained Age: 22.0 ± 5.0 yr Weight: 86.0 ± 5.5 kg Height:1.93 ± 0.10 m TE: 8.0 ± 2.9 yr	1) CON: 7 min tactical instructions jogging; dribbling; shooting the ball 2) EXP-a: jogging; dribbling; shooting the ball; sprint; shuffles; 5 vertical jumps (60; 70; 80; 90; 95% ME) 3) EXP-b: 2) + stabilization exercises	Pre-half time RWU Post-half time RWU	1) CMJ ↓* 2) and 3) CMJ ↔
González-Devesa et al. (2022)	10F highly trained Age: 13.7 ± 0.8 yr Weight: 55.4 ± 6.9 kg Height:1.63 ± 0.50 m	1) CON: 10min passive rest 2) EXP: 6min passive rest + 5×14m sprint + shooting wheel for 2 min	Post-WU Pre-half time RWU Post-half time RWU Post-Q3	1) all CMJ ↔ 2) all CMJ ↔ Post-half time RWU RPE 2) > 1) *
González-Devesa et al. (2023)	10F highly trained Age: 14.2 ± 1.6 yr Weight: 54.0± 14.0 kg Height:1.62 ± 0.11 m	1) CON: 3 min basketball-specific shooting 2) EXP: 1 min single-leg bouncing (8×2 bounds to each leg) +2min shooting at the basket	Post-WU Pre-half time RWU Post-half time RWU Post-Q4	1) all CMJ ↔ 2) all CMJ ↔ Post-half time RWU RPE 2) vs 1) ↔
Koutsouridis et al. (2024)	13M highly trained Age: 20.5 ± 1.5 yr Weight: 81.4± 6.4 kg Height:1.82 ± 0.7 m TE: 9.1 ± 3.2 yr	1) CON: 15min passive rest 2) EXP: 11min passive rest + 3min cycling at 40%Vo2max + 1 min rest	Pre-half time RWU Post-half time RWU	1) CMJ, BT, COD, and RPE ↓* 2) CMJ, BT, COD, and RPE ↔
Flórez-Gil et al. (2025b)	15F highly trained age: 16.7 ± 0.6 yr weight: 61.7± 8.7 kg height:1.68 ± 0.07 m TE: 4.2 ± 1.1 yr	1) CON: 15min passive rest 2) EXP-a: one-foot low skipping ladder; Multidirectional multi-jumps; Abalakov jumps 3) EXP-b: High knees; Butt kicks; Forward and lateral lunges; Forward leg swings 4) EXP-c: Players were draped with towels over their heads and lower limbs (2 min exercise and 1 min rest, repeated for 15 min)	Post-WU Pre-Q1	1) CMJ, DJ, and COD ↓* 2) CMJ, DJ, COD ↔ 3) CMJ ↓*; DJ and COD ↔ 4) CMJ ↓*; DJ and COD ↔
Koutsouridis et al. (2025)	12M highly trained Age: 20.3 ± 1.3 yr Weight: 82.2 ± 5.9 kg Height:1.84 ± 0.07 m TE: 9.5 ± 2.9 yr	1) CON: 15min passive rest 2) EXP-a: 11min passive rest + 3min cycling at 80%Vo2max + 1 min rest	Pre-half time RWU Post-half time RWU	1) CMJ, BT, COD, and RPE ↓* 2) RPE ↔; CMJ, BT, and COD ↓*
Yang et al. (2025)	12M Highly trained Age: 20.5 ± 1.6 yr Weight: 84.0 ± 7.8 kg Height:1.86 ± 0.07 m TE: 8.0 ± 2.3 yr	1) CON: 15 min passive rest 2) EXP-a:10 min rest + 4 min CSE (stable) 3) EXP-b: 10 min rest + 4 min CSE (unstable) CSE: superman, rocking side-to-side plank, active side plank, mountain climbers, and active single leg bridge	Post-WU Post-half time RWU	1) COD, CMJ, and IMTP-PF ↓*; SJ, BT, and RPE ↔ 2) IMTP-PF ↓*; CMJ, SJ, COD, BT, and RPE ↔ 3) IMTP-PF ↓*; CMJ, SJ, COD, BT, and RPE ↔

M, Male; F, Female; yr, year; TE, training experience; CON, control; EXP, experiment; ME, maximum effort; RWU, re-warm-up; CMJ, countermovement jump; WU, warm-up; RPE, rating of perceived exertion; Q1, the first quarter; Q3, the third quarter; Q4, the fourth quarter; BT, body temperature; COD, change of direction; DJ, drop jump; CSE, core strength exercise; IMTP-PF, isometric midthigh pull peak force; SJ, squat jump.

### 3.2 Risk of bias assessment

Of the included studies, six (85.7%) were rated as having *some concerns* regarding overall risk of bias (González-Devesa et al., 2022; González-Devesa et al., 2023; Koutsouridis et al., 2024, 2025; Flórez-Gil et al., 2025b; Yang et al., 2025), while one (14.3%) were classified as *high risk* (Pociūnas et al., 2018). All studies were rated *low risk* for deviations from intended interventions and missing outcome data. As none of the studies were prospectively registered, the risk of selective reporting remains *unclear*, and thus rated as *some concerns* for all studies (See **Appendix 3** of the **Supplementary Materials**).

### 3.3 The acute effect of RWU on jump, COD, and IMTP peak force

**Figure 2:**
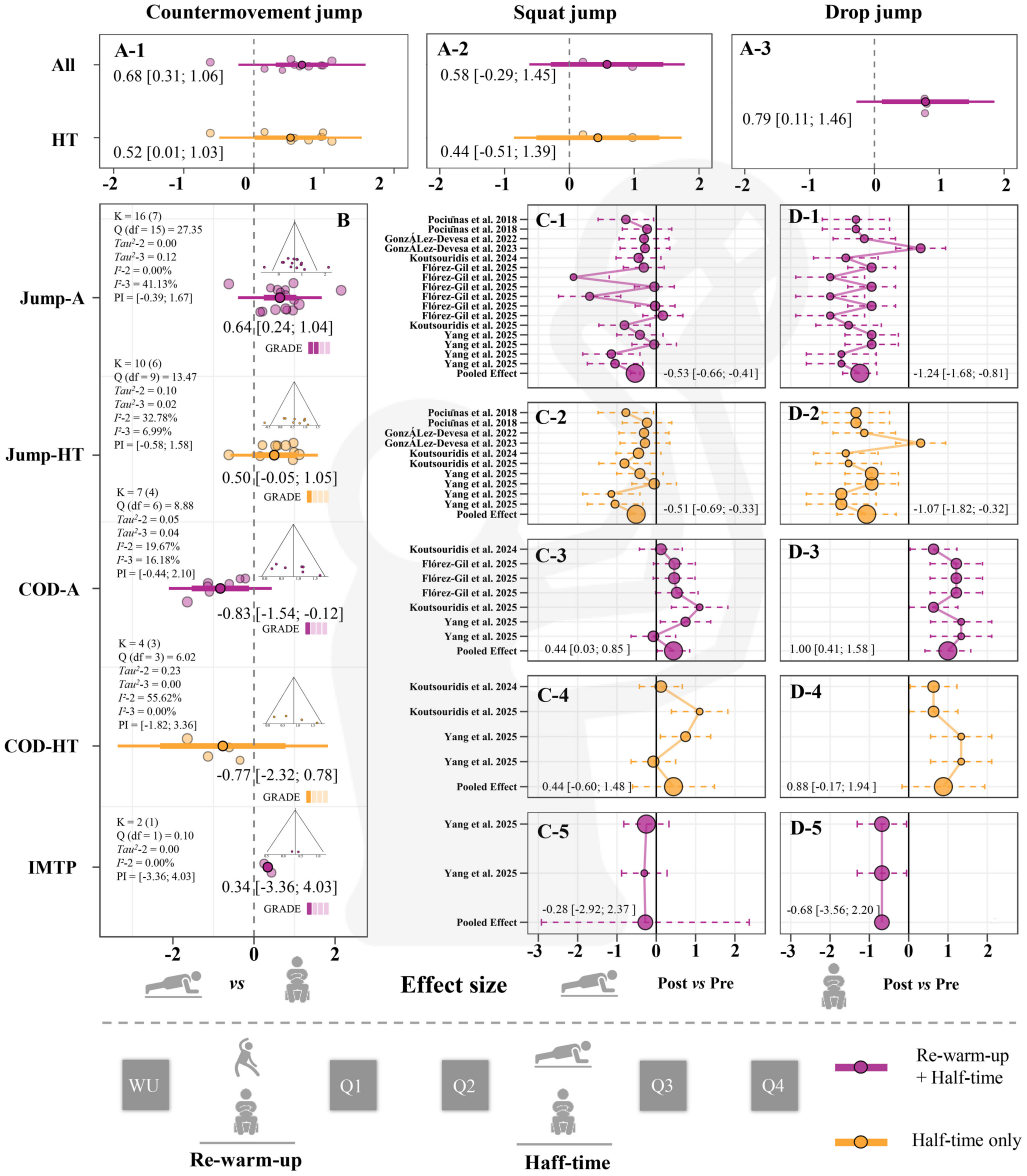
Effects of re-warm-up (between- and within-group comparisons) on physical performance in basketball players. **(A)** Subgroup analysis; **(B)** Effects of re-warm-up on jumping performance, COD, and IMTP peak force; **(C)** Effects of halftime re-warm-up on jumping performance, COD, and IMTP peak force; **(D)** Effects of the control condition on jumping performance, COD, and IMTP peak force. HT, halftime; A, all; COD, change of direction; IMTP, isometric mid-thigh pull.

When all RWU conditions were considered, RWU significantly improved jumping performance (SMD = 0.64, 95% CI [0.24, 1.04], GRADE = Low, **Figure 2B**) and COD performance (SMD = -0.83, 95% CI [-1.54, -0.12], GRADE = Low, **Supplementary Table 1**) compared with the control condition, but had no significant effect on IMTP peak force (SMD = 0.34, 95% CI [-3.36, 4.05], GRADE = Very low). However, after removing the study by Flórez-Gil et al. (2025b), the effects on both jumping performance (SMD = 0.50, 95% CI [-0.05, 1.05], GRADE = Very low) and COD performance (SMD = -0.77, 95% CI [-2.32, 0.78], GRADE = Very low) were no longer significant.

Subgroup analyses for jumping performance showed that, both with and without the study by Flórez-Gil et al. (2025b), RWU significantly increased CMJ height (all studies: SMD = 0.68, 95% CI [0.31, 1.06]; after removal: SMD = 0.52, 95% CI [0.01, 1.03], **Figure 2A**), but had no significant effect on SJ height (all studies: SMD = 0.58, 95% CI [-0.29, 1.45]; after removal: SMD = 0.44, 95% CI [-0.51, 1.39]). When the RWU was performed after the initial warm-up, DJ height was significantly improved compared with the control condition (SMD = 0.79, 95% CI [0.11, 1.46]). Notably, no significant differences were observed among the three jump outcomes (*p* = 0.65 or 0.53).

Further within-group comparison analyses indicated that, regardless of whether the study by Flórez-Gil et al. (2025b)was removed, both the RWU group (all studies: SMC = -0.53, 95% CI [-0.66, -0.41]; after removal: SMC = -0.51, 95% CI [-0.69, -0.33], **Figure 2C**) and the control group (all studies: SMC = -1.24, 95% CI [-1.68, -0.81]; after removal: SMC = -1.07, 95% CI [-1.82, -0.32], **Figure 2D**) showed significant reductions in jump height. When the study by Flórez-Gil et al. (2025b) was included, COD performance significantly declined in both the RWU group (SMC = 0.44, 95% CI [0.03, 0.85]) and the control group (SMC = 1.00, 95% CI [0.41, 1.58]); however, after removing this study, the differences were no longer significant (RWU: SMC = 0.44, 95% CI [-0.60, 1.48]; control: SMC = 0.88, 95% CI [-0.17, 1.94]). Only one study reported IMTP peak force, and the results were not significant in either the RWU group (SMC = -0.28, 95% CI [-2.92, 2.37]) or the control group (SMC = -0.68, 95% CI [-3.56, 2.20]).

### 3.4 The acute effect of RWU on BT and RPE

**Figure 3:**
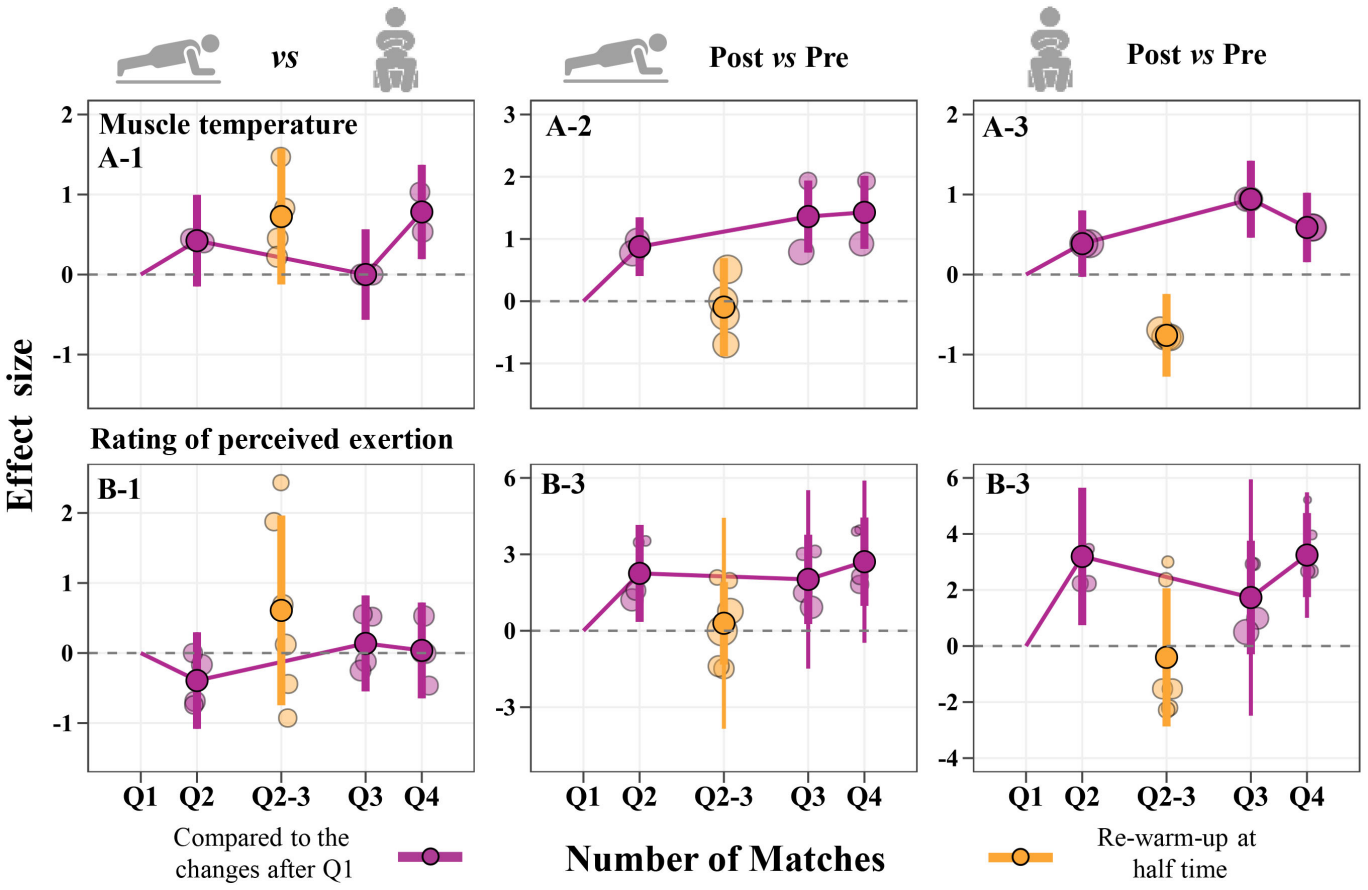
Effects of halftime re-warm-up on body temperature **(A)** and rating of perceived exertion **(B)** in basketball players. 1: between-group comparison; 2: within-group comparison in the re-warm-up condition; 3: within-group comparison in the control condition.

Compared with the control condition, halftime RWU showed no differences on BT (SMD = 0.72; 95% CI [-0.12, 1.57], **Figure 3A**) or RPE (SMD = 0.61; 95% CI [-0.75, 1.96], **Figure 3B**). When compared with values after the initial warm-up, only BT at the end of the fourth quarter was significantly higher than after the first quarter (SMD = 0.78; 95% CI [0.19, 1.37]), whereas no other significant differences were observed for RPE or BT across time points (*p* > 0.05).

Within-group comparison analyses showed that the halftime RWU group exhibited no significant changes in BT (SMC = -0.09; 95% CI [-0.88, 0.69]) or RPE (SMC = 0.29; 95% CI [-1.33, 1.91]). In contrast, the halftime control group showed a significant reduction in BT (SMC = -0.76; 95% CI [-1.28, -0.24]), but no significant change in RPE (SMC = -0.40; 95% CI [-2.87, 2.06]). Compared with post–initial warm-up values, both the RWU and control groups showed significant increases in BT and RPE at the end of the second, third, and fourth quarters (*p* < 0.05). However, in the control group, the change in BT at the end of the second quarter did not reach statistical significance (*p* = 0.07), and the change in RPE at the end of the third quarter was also not significant (*p* = 0.07).

### 3.5 Percentage of individual improvements in countermovement jump and COD

**Figure 4:**
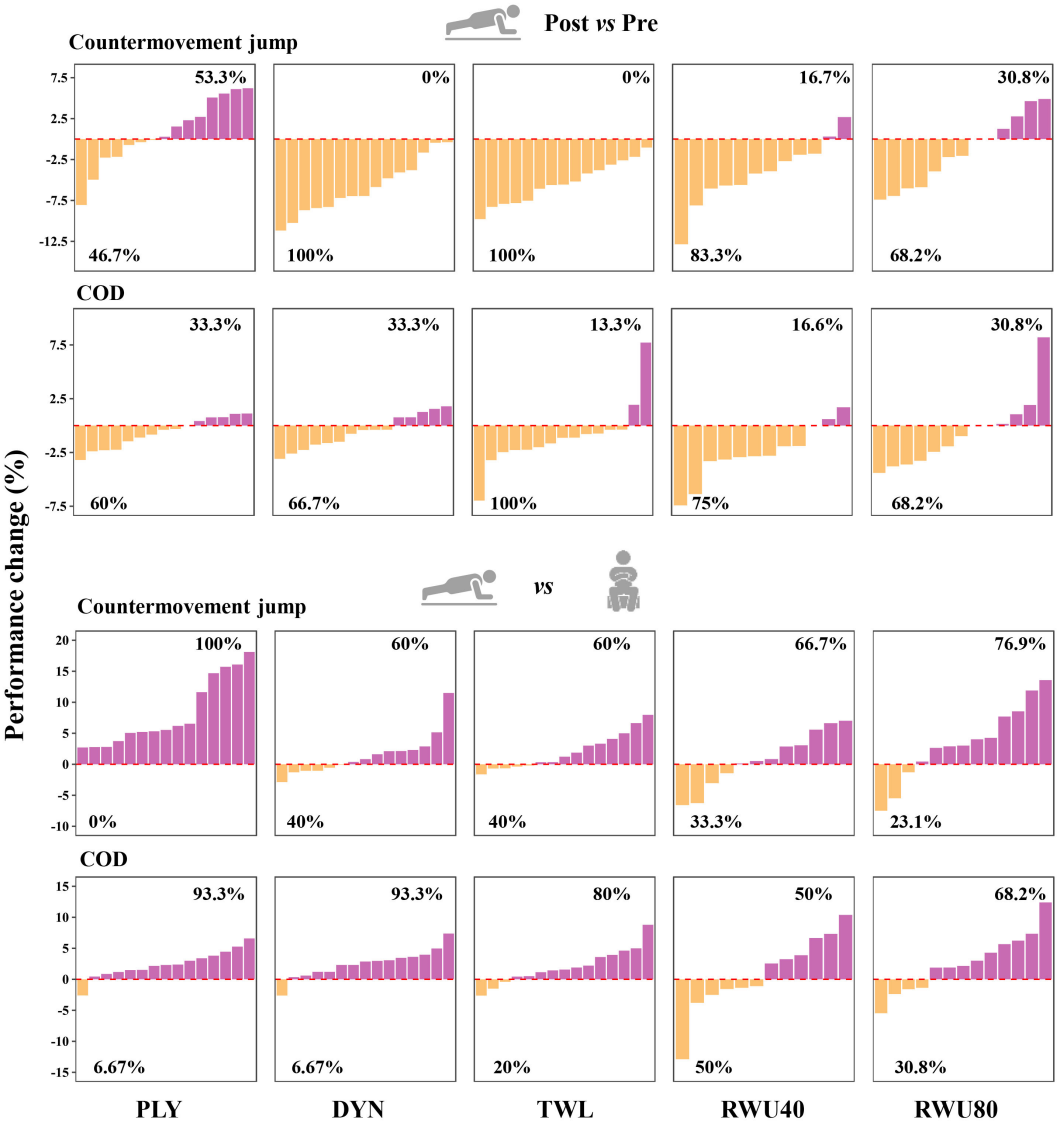
Individualized analyses based on three studies by (Koutsouridis et al., 2024, 2025) and (Flórez-Gil et al., 2025b) PLY: one-foot low skipping ladder; multidirectional multi-jumps; and Abalakov jumps. DYN: high knees; butt kicks; forward and lateral lunges; and forward leg swings. TWL: players were draped with towels over their heads and lower limbs. RWU40: 3-min cycling at 40% VO_2_max. RWU80: 1-min cycling at 80% VO_2_max. Note. In the study by Flórez-Gil, the protocol consisted of 2-min exercise followed by 1-min rest, repeated for 15 min. COD, change of direction.

When only pre–post changes following the RWU were considered, the plyometric jump group used by Flórez-Gil et al. (2025b)showed the highest proportion of individual improvements among basketball players, with 53.3% improving in CMJ and 33.3% improving in COD (**Figure 4**). When compared with the control condition, the proportion of improvements in the plyometric group increased further, reaching 100% for CMJ and 93.3% for COD. During halftime RWU, the 1-min cycling protocol at 80% of maximal oxygen uptake used by Koutsouridis et al. (2025) resulted in 30.8% of players improving both CMJ and COD compared with pre-halftime values. When compared with the control condition, the proportion of players showing improvements increased to 76.9% for CMJ and 68.2% for COD (**Figure 4**).

## 4 Discussion

This study examined the effects of RWU on jumping performance (e.g., CMJ, SJ, and DJ), COD performance, IMTP peak force, BT, and RPE in basketball players using an individual participant data meta-analysis. The overall results indicated that, when both post–initial warm-up and halftime RWU were considered together, RWU significantly improved jumping and COD performances compared with the control condition, while showing no differences in IMTP, BT, or RPE. However, after removing the study by Flórez-Gil et al. (2025b), halftime RWU alone no longer produced significant improvements in jumping or COD performance. When within-group comparisons were examined, the control group showed greater declines in jumping performance, COD performance, and BT than the RWU group. Therefore, these improvements mainly indicate that RWU better preserved performance compared with the control, rather than producing a true performance gain. Moreover, based on the studies with available individual data (Koutsouridis et al., 2024, 2025; Flórez-Gil et al., 2025b), the plyometric protocol used by Flórez-Gil et al. (2025b) and the 1-min cycling at 80% of maximal oxygen uptake used by Koutsouridis et al. (2025) produced improvements in CMJ and COD in a large proportion of participants, with even greater proportions when compared with the control condition. From a practical perspective, the plyometric protocol described by Flórez-Gil et al. (2025b) may be suitable for non-starting players (**Figure 4**), whereas a 1-min cycling protocol at 80% of maximal oxygen uptake may be considered for halftime RWU (**Figure 4**). However, given the limited number of studies, the optimal RWU strategy remains unclear.

The current results showed that the RWU group significantly improved jumping (SMD = 0.64) and COD performance (SMD = −0.83) compared with the control condition. This effect was largely driven by the greater performance declines observed in the control group (SMC = −1.24 or 1.00) than in the RWU group (SMC = −0.53 or 0.44). Thus, the primary benefit of RWU may lie in attenuating performance declines rather than further enhancing performance. Furthermore, after removing the study by Flórez-Gil et al. (2025b) halftime RWU alone showed no significant effects on jumping or COD. This may suggest that, under conditions of prior fatigue (i.e., after two quarters of play), the effectiveness of RWU is reduced (Chiu and Barnes, 2003; Zhu et al., 2026). In contrast, RWU following the initial warm-up appears more likely to enhance performance. This interpretation is supported by the subgroup analysis of jumping performance, in which the SMD for CMJ and SJ decreased from 0.68 and 0.58 to 0.52 and 0.44, respectively, after excluding the Flórez-Gil et al. (2025b) study. Another important piece of evidence is that the RWU group in Flórez-Gil et al. (2025b) showed a significant improvement in DJ performance compared with the control group. Together, these findings suggest that RWU may produce greater performance benefits when prior fatigue is absent, whereas the effects may be smaller when fatigue is already present (Chiu and Barnes, 2003; Zhu et al., 2026). However, this remains speculative, as fatigue markers were not directly measured in the included studies.

The individual-level results also support the potential influence of prior fatigue on the effectiveness of RWU. In the three conditions examined by Flórez-Gil et al. (2025b) the proportion of players showing improvements in CMJ ranged from 60% to 100%, and in COD from 80% to 93.3% when compared with the control condition. In contrast, the two studies by Koutsouridis et al (Koutsouridis et al., 2024, 2025) reported improvement rates of 66.7% and 76.9% for CMJ, and 50% and 68.2% for COD. Overall, RWU implemented immediately after the initial warm-up may be more effective than halftime RWU in enhancing physical performance, likely due to the combined potentiation effects of the initial warm-up and RWU, which can optimize neuromuscular readiness, a benefit that is less pronounced when RWU is performed after halftime. Specifically, this enhancement likely involves multiple mechanisms, including increased neural activation (Bishop, 2003), post-activation potentiation (Blazevich and Babault, 2019), post-activation performance enhancement (Xu et al., 2025a), elevated muscle temperature (Bishop, 2003), and improved muscle contractile properties (Tillin and Bishop, 2009). These mechanisms collectively influence the balance between fatigue and performance enhancement (Zhu et al., 2026). In contrast, RWU performed at halftime is conducted on a background of accumulated fatigue, whereas RWU following the initial warm-up is associated with a lower fatigue state (Rassier and MacIntosh, 2000). This difference in fatigue levels may be a key factor explaining why performance improvements are more likely to occur when RWU is implemented immediately after the initial warm-up.

Different strategies may also lead to different proportions of responders. After the initial warm-up, the plyometric protocol appeared to be an effective strategy. In contrast, during halftime, both 1-min cycling at 80% of maximal oxygen uptake and 3-min cycling at 40% of maximal oxygen uptake were associated with performance declines in some players. This highlights the substantial individual variability in response to RWU, where the balance between performance enhancement and fatigue determines the ultimate outcome (Zhu et al., 2026). Although González-Devesa et al (González-Devesa et al., 2022; González-Devesa et al., 2023). reported no significant differences between RWU and control conditions in second-half running distance or mean session velocity, the individual-level results indicate that adding a RWU under conditions of accumulated fatigue may produce negative responses in some players. Therefore, in real-game settings, the plyometric protocol described by Flórez-Gil et al. (2025b)may be effective for non-starting players, but it is important not to generalize this outcome to the entire squad. Halftime RWU strategies should be individualized, and in some cases, no RWU may be preferable (Tomaras and MacIntosh, 2011).

Regarding BT and RPE, halftime RWU generally helped maintain BT in basketball players, while showing no significant differences in RPE compared with the control condition. It should be noted that BT in the included studies was generally measured using tympanic (ear) thermometers, reflecting core BT rather than local muscle temperature. However, direct assessments of muscle temperature (e.g., intramuscular measurements) are invasive and difficult to implement in applied sport settings, and thus tympanic temperature is often used as a practical surrogate despite its limitations. Regarding RPE, the lack of significant differences may be explained by several factors, including the relatively short duration and/or low intensity of RWU protocols. Additionally, the limited number of studies and small sample sizes may have reduced the sensitivity to detect meaningful changes. Therefore, these findings should be interpreted with caution.

The main limitation of this study is the small number of included studies (k = 7) and the wide confidence intervals, which may reduce the precision of the pooled estimates. The overall findings should therefore be considered preliminary. Although we attempted to address this limitation by obtaining individual participant data, raw data were available from only three studies (Koutsouridis et al., 2024, 2025; Flórez-Gil et al., 2025b). This limited our ability to explore the effectiveness of RWU at the full individual level. In addition, some comparisons were based on a single study, such as IMTP peak force and DJ performance. Therefore, the present study should be considered primarily quantitative and exploratory in nature. A further limitation is that the existing studies have not extensively investigated actual basketball skill performance (e.g., shooting, passing, or game-specific technical actions). Most outcomes were limited to physical performance measures, which may not fully reflect the practical impact of RWU strategies during real match situations. Moreover, despite the limited number of studies, the inter-individual variability observed suggests that RWU strategies should not be generalized; instead, they must be tailored to the specific context and the unique physical profile of each athlete. Future research should therefore examine the effects of RWU on basketball-specific skill performance and consider individual responses to provide more ecologically valid and practically relevant evidence.

## 5 Conclusions

Preliminary findings from this small sample suggest that both post–initial warm-up and halftime RWU may better preserve jumping and COD performance and help maintain BT in basketball players compared with a control condition, while showing similar effects on IMTP peak force and RPE. However, these results were largely driven by greater performance declines in the control group than in the RWU group. Thus, RWU may offer advantages for maintaining physical performance and BT.

In addition, RWU performed after the initial warm-up may be more effective than halftime RWU in improving jumping and COD performance compared with the control condition; however, this finding should be interpreted with caution and warrants further investigation due to the limited evidence base.

From a practical perspective, the plyometric protocol described by Flórez-Gil et al. (2025b) may be suitable for non-starting players, whereas a 1-min cycling protocol at 80% of maximal oxygen uptake may be considered for halftime RWU. However, given the limited number of studies, the optimal RWU strategy remains unclear.

## Data Availability

The datasets presented in this study can be found in online repositories. The names of the repository/repositories and accession number(s) can be found in the article/**Supplementary Material**.
