# Supplementary material for: Acute effects of re-warm-up on physical performance, muscle temperature, and perceived exertion in basketball players: a systematic review and meta-analysis with individual participant data
Source: Front Physiol. 2026 Jul 8;17:1821278. doi: 10.3389/fphys.2026.1821278 (PMC13388047; doi:10.3389/fphys.2026.1821278)
Supplement: Supplementary file 1 [file SupplementaryFile1.docx]

**Acute Effects of Re-Warm-Up on Physical Performance, Muscle Temperature, and Perceived Exertion in Basketball Players: A Systematic Review and Meta-Analysis with Individual Participant Data**

**Yu-Feng Yang ^1*^, Enrique Flórez-Gil^2^, Christos Koutsouridis^3^, Yu-Ming Zhong^4,5^ Nuno Mateus^6,7^, Eduardo Abade^6,7,8^, Ching-Feng Cheng^9,10^, Mengde Lyu^11^, Kai Xu^4,5^**

^1^School of Physical Education, Shanghai University of Sport, No. 200, Henren Road, Shanghai, 200438, China

^2^Faculty of Physical Activity and Sports Sciences, VALFIS Research Group, Institute of Biomedicine (IBIOMED), Universidad de León, León, Spain

^3^Aristotle University of Thessaloniki, School of Physical Education & Sport Science, Laboratory of Evaluation of Human Biological Performance, Thessaloniki, GREECE

^4^School of Medical and Health Sciences, Centre for Human Performance, Edith Cowan University, Joondalup, Australia

^5^School of Athletic Performance, Shanghai University of Sport, Shanghai, China

^6^Research Center in Sports Sciences, Health Sciences and Human Development, CIDESD, Elite Research Community, Vila Real, Portugal;

^7^Department of Sports Science, Exercise and Health, School of Life Sciences and Environment, University of Trás-os-Montes and Alto Douro, Vila Real, Portugal;

^8^Portugal Football School, Portuguese Football Federation, Oeiras, Portugal

^9^Department of Sport and Kinesiology, National Taiwan Normal University, Taipei, Taiwan;

^10^Sports Performance Lab, Department of Sport and Kinesiology, National Taiwan Normal University, Taipei, Taiwan

^11^School of Biomedical Science and Health, Royal Melbourne Institute of Technology University, Melbourne, Australia

*** Correspondence:**Yu-Feng Yang

[yyfeng996@163.com](mailto:yyfeng996@163.com);

**Contents**

|  |  |
| --- | --- |
| **Appendix 1: PRISMA-IPD Checklist** | **3-5** |
| **Appendix 2: Search strategy** | **6** |
| **Appendix 3: Risk of bias assessments** | **6** |
| **Appendix 4: GRADE assessment** | **7** |

**Electronic Supplementary Materials**

# **Appendix 1: PRISMA-IPD Checklist**

| **PRISMA-IPD**  **Section/topic** | **Item**  **No** | **Checklist item** | **Reported on page** |
| --- | --- | --- | --- |
| **Acute Effects of Re-Warm-up on Athletic Performance, Perceived Exertion, and Muscle Temperature in Basketball Players: A Systematic Review and Meta-Analysis with Individual Participant Data** | | | |
| Title | 1 | Identify the report as a systematic review and meta-analysis of individual participant data. | Title |
| **Abstract** | | | |
| Structured summary | 2 | Provide a structured summary including as applicable: | Abstract |
|  |  | **Background**: state research question and main objectives, with information on participants, interventions, comparators and  outcomes. |  |
|  |  | **Methods**: report eligibility criteria; data sources including dates of last bibliographic search or elicitation, noting that IPD were  sought; methods of assessing risk of bias. |  |
|  |  | **Results**: provide number and type of studies and participants identified and number (%) obtained; summary effect estimates for main outcomes (benefits and harms) with confidence intervals and measures of statistical heterogeneity. Describe the direction  and size of summary effects in terms meaningful to those who would put findings into practice. |  |
|  |  | **Discussion:** state main strengths and limitations of the evidence, general interpretation of the results and any important  implications. |  |
|  |  | **Other:** report primary funding source, registration number and registry name for the systematic review and IPD meta-analysis. |  |
| **Introduction** | | | |
| Rationale | 3 | Describe the rationale for the review in the context of what is already known. | Introduction |
| Objectives | 4 | Provide an explicit statement of the questions being addressed with reference, as applicable, to participants, interventions, comparisons, outcomes and study design (PICOS). Include any hypotheses that relate to particular types of participant-level  subgroups. | Introduction |
| **Methods** | | | |
| Protocol and registration | 5 | Indicate if a protocol exists and where it can be accessed. If available, provide registration information including registration number and registry name. Provide publication details, if applicable. | Methods |
| Eligibility criteria | 6 | Specify inclusion and exclusion criteria including those relating to participants, interventions, comparisons, outcomes, study design and characteristics (e.g. years when conducted, required minimum follow-up). Note whether these were applied at the study or individual level i.e. whether eligible participants were included (and ineligible participants excluded) from a study that included a wider population than specified by the review inclusion criteria. The rationale for criteria should be stated. | Methods |
| Identifying  studies - | 7 | Describe all methods of identifying published and unpublished studies including, as applicable: which bibliographic databases  were searched with dates of coverage; details of any hand searching including of conference proceedings; use of study registers | Methods |
| Exploration of variation in effects | A2 | If applicable, describe any methods used to explore variation in effects by study or participant level characteristics (such as estimation of interactions between effect and covariates). State all participant-level characteristics that were analysed as potential effect modifiers, and whether these were pre-specified. | Methods |
| Risk of bias across studies | 15 | Specify any assessment of risk of bias relating to the accumulated body of evidence, including any pertaining to not obtaining IPD for particular studies, outcomes or other variables. | Methods |
| Additional analyses | 16 | Describe methods of any additional analyses, including sensitivity analyses. State which of these were pre-specified. | Methods |
| **Results** | | | |
| Study selection and IPD obtained | 17 | Give numbers of studies screened, assessed for eligibility, and included in the systematic review with reasons for exclusions at each stage. Indicate the number of studies and participants for which IPD were sought and for which IPD were obtained. For those studies where IPD were not available, give the numbers of studies and participants for which aggregate data were available. Report reasons for non-availability of IPD. Include a flow diagram. | Results |
| Study characteristics | 18 | For each study, present information on key study and participant characteristics (such as description of interventions, numbers of participants, demographic data, unavailability of outcomes, funding source, and if applicable duration of follow-up). Provide (main) citations for each study. Where applicable, also report similar study characteristics for any studies not providing IPD. | Results |
| IPD integrity | A3 | Report any important issues identified in checking IPD or state that there were none. | Results |
| Risk of bias within studies | 19 | Present data on risk of bias assessments. If applicable, describe whether data checking led to the up-weighting or down- weighting of these assessments. Consider how any potential bias impacts on the robustness of meta-analysis conclusions. | Results |
| Results of individual studies | 20 | For each comparison and for each main outcome (benefit or harm), for each individual study report the number of eligible participants for which data were obtained and show simple summary data for each intervention group (including, where applicable, the number of events), effect estimates and confidence intervals. These may be tabulated or included on a forest plot. | Results |
| Results of syntheses | 21 | Present summary effects for each meta-analysis undertaken, including confidence intervals and measures of statistical heterogeneity. State whether the analysis was pre-specified, and report the numbers of studies and participants and, where applicable, the number of events on which it is based. | Results |
|  |  | When exploring variation in effects due to patient or study characteristics, present summary interaction estimates for each characteristic examined, including confidence intervals and measures of statistical heterogeneity. State whether the analysis was pre-specified. State whether any interaction is consistent across trials. |  |
|  |  | Provide a description of the direction and size of effect in terms meaningful to those who would put findings into practice. |  |
| Risk of bias across studies | 22 | Present results of any assessment of risk of bias relating to the accumulated body of evidence, including any pertaining to the | Results |
|  |  | availability and representativeness of available studies, outcomes or other variables. | Results |
| Additional analyses | 23 | Give results of any additional analyses (e.g. sensitivity analyses). If applicable, this should also include any analyses that incorporate aggregate data for studies that do not have IPD. If applicable, summarise the main meta-analysis results following the inclusion or exclusion of studies for which IPD were not available. | Results |
| **Discussion** | | | |
| Summary of evidence | 24 | Summarise the main findings, including the strength of evidence for each main outcome. | Discussion |
| Strengths and limitations | 25 | Discuss any important strengths and limitations of the evidence including the benefits of access to IPD and any limitations arising from IPD that were not available. | Discussion |
| Conclusions | 26 | Provide a general interpretation of the findings in the context of other evidence. | Discussion |
| Implications | A4 | Consider relevance to key groups (such as policy makers, service providers and service users). Consider implications for future research. | Discussion |
| **Funding** | | | |
| Funding | 27 | Describe sources of funding and other support (such as supply of IPD), and the role in the systematic review of those providing such support. | Funding |

# **Appendix 2: Search strategy**

| **Database** | **Specificities of the database** | **Search strategy** |
| --- | --- | --- |
| PubMed | All field | (re warm up) AND (basketball OR non started OR half time) |
| Web of Sceience | Topic | (re warm up) AND (basketball OR non started OR half time) |
| SPORTDiscus | All fields | (re warm up) AND (basketball OR non started OR half time) |

# **Appendix 3: Risk of bias assessments**

**Figure S1.** Risk bias assessment.

# **Appendix 4: GRADE assessment**

| **Table S1** GRADE assessment for the certainty of evidence | | | | | | | | | |
| --- | --- | --- | --- | --- | --- | --- | --- | --- | --- |
| Certainty assessment | | | | | |  | No. of participants | | Certainty |
| No. of  studies | Risk of  bias | Inconsistency | Indirectness | Imprecision | Publication  bias |  | Re-warm-up | Absolute (95% CI) |  |
| EXP vs. CON-all (jump) | | | | | | | | | |
| 7 | Not serious | Not serious | Not serious | Serious  -1 | Serious  **-1** |  | 82 | SMD 0.64 (0.24 to 1.04) | ⨁⨁○○ Low |
| EXP vs. CON-half-time (jump) | | | | | | | | | |
| 6 | Not serious | Not serious | Not serious | Serious  -2 | Serious  -1 |  | 67 | SMD 0.50 (-0.05 to 1.05) | ⨁○○○ Very Low |
| EXP vs. CON-all (change of direction) | | | | | | | | | |
| 4 | Not serious | Not serious | Not serious | Serious  -1 | Serious  -1 |  | 52 | SMD -0.83 (-1.54 to -0.15) | ⨁⨁○○ Low |
| EXP vs. CON-half-time (change of direction) | | | | | | | | | |
| 3 | Not serious | Serious  -1 | Not serious | Serious  -2 | Serious  -1 |  | 39 | SMD -0.77 (-2.32 to 0.78) | ⨁○○○ Very Low |
| EXP vs. CON-all (isometric midthigh pull peak force) | | | | | | | | | |
| 1 | Not serious | Not serious | Not serious | Serious  -2 | Serious  -1 |  | 12 | SMD 0.34 (-3.36 to 4.03) | ⨁○○○ Very Low |
| EXP, experiment; CON control; CI confident interval; SMD standard mean difference. | | | | | | | | | |
